# Temperature, Time, and Interactions between Them in Relation to Colour Parameters of Black Poplar (*Populus nigra* L.) Thermally Modified in Nitrogen Atmosphere

**DOI:** 10.3390/ma15030824

**Published:** 2022-01-21

**Authors:** Olga Bytner, Michał Drożdżek, Agnieszka Laskowska, Janusz Zawadzki

**Affiliations:** The Institute of Wood Sciences and Furniture, 159 Nowoursynowska St., 02-776 Warsaw, Poland; olga_bytner@sggw.edu.pl (O.B.); agnieszka_laskowska@sggw.edu.pl (A.L.); janusz_zawadzki@sggw.edu.pl (J.Z.)

**Keywords:** colour, lightness, nitrogen atmosphere, poplar, modification temperature, modification time

## Abstract

Thermal modification of wood in nitrogen atmosphere permits its usability value to be improved. The aim of the research was to determine the impact of technological modification parameters at different levels on the colour of black poplar (*Populus nigra* L.). Black poplar was thermally modified in nitrogen atmosphere at a range of temperatures from 160 °C to 220 °C, at times between 2 h and 8 h. The parameters of wood colour were measured according to the CIE L*a*b* colour space model. The changes in a* and b* had a non-linear profile. The maximum value of a* for black poplar wood was achieved after a modification at the temperature of 200 °C, while the maximum value of the b* parameter was achieved after modification at 190 °C. Colour changes in the ΔE of black poplar after modification at 160 °C and 170 °C were similar, and the dynamics of changes increased after modification at the temperature of 180 °C. The highest value of ΔE, around 40, was observed after modification at the temperature of 220 °C and time of 8 h. There were no statistically significant differences between the ΔE for radial and tangential sections. Statistical analysis showed that modification temperature was responsible for the variability of the L* value in 90%, and in ca. 70% for the changes in parameters a* and b*. The influence of the modification time on the colour parameters was minor—below 4%. The influence of the interaction between modification temperature and time on the colour parameters was below 10%. As a consequence, in the case of ΔE of black poplar wood, the influence of temperature was at a level of ca. 80%. On the other hand, the influence of time and the interaction between temperature and time of modification was low—below 3%.

## 1. Introduction

The development of the wood industry depends, to a large extent, on the implementation of innovative solutions in the scope of production technology. One of the methods to achieve those goals is through processes of wood modification, mostly thermal processes. Thermal modification is an ecological process [1,2]. Wood that undergoes such modification does not become toxic but in the first phase after modification process releases some organic compounds such as furfural or acetic acid [3]. Thermal modification is used to improve the aesthetical and usability properties of local wood species or to increase their resistance to destructive environmental factors [4]. As a result, the commercial importance of less competitive wood species can increase in the market [5], which in turn can lead to a more rational management of forest resources. Moreover, in recent years, a tendency to substitute wood of high mechanical properties with lower quality wood can be seen. The development of such modifications has been accelerated by a growing ecological awareness, high prices, and difficult access to tropical wood. Modified wood can replace the tropical species due to the good functional properties and dark colour achieved as a result of modification [6].

There are many methods of thermal modification that can be carried out in different kinds of environments: vacuum, air, nitrogen, or oil [7,8]. Currently, one of the most popular methods of modification is the ThermoWood^®^ method, which is being carried out in superheated steam at atmospheric pressure [9]. Studies on wood modification and its effects are also carried out for nitrogen atmosphere treatment [10]. Results published in the literature indicate that nitrogen modification is less destructive compared to modification in water vapour. The mass loss of black poplar modified in superheated steam for 2 h at the temperature of 160 °C, 190 °C, and 220 °C amounted to 3%, 4%, and 12%, correspondingly, while for nitrogen modification these values amounted to 1%, 1%, and 7%, respectively [11,12].

Poplar is one of the most efficient trees in terms of sustainability. Poplar, mainly the cultivar “Hybrid 275”, from plantation crops, is used for energy purposes or as a source of raw material for the wood-based panels sector. Black poplar wood is used mainly as a resource for the cellulose industry and as a fuel material [12]. Poplar is one of the fastest growing trees in the world. In Europe, one cubic meter of lumber can be produced, on average, in 15 years. Compared to other trees such as oak, that take more than 100 years, poplars grow very quickly [13]. With the growing worldwide shortage of forest resources, especially precious wood, a large number of fast-growing species are widely cultivated in order to meet the global timber demand. The European survey “EUwood” showed that wood deficiency could reach 200 million m^3^ and 300 million m^3^ in 2025 and 2030, respectively [14,15].

Fast-growing wood with physical or chemical modification not only increases the added value of fast-growing wood, but also plays a role in the protection of precious tree species resources [16]. Black poplar plays an important role in the wood industry, and the advantage of this species consists in its plantation potential resulting from a fast increase of biomass [17]. This has been confirmed by the inclusion of black poplar in the EN 13556:2003 standard [18] concerning wood terminology in the European timber trade, where it has the four-letter code PONG. It lacks a clearly formed heartwood (strongly saturated with non-structural compounds). Moreover, it has a drab greyish colour and monotonous pattern. Often on the basis of pathological factors (fungal penetration and their destructive activity) or under the influence of climatic factors [19], poplar wood can show a “false heartwood” with sometimes a very dark and non-homogeneous colour. Due to the above-mentioned drawbacks, the wood of black poplar is rarely used for the production of solid wood elements. Usually, it serves as a source of cellulose for the paper industry, for the production of derivative wood materials, or as a firewood fuel. Thermal modification of black poplar enhances its properties and thus can extend its range of applications.

After thermal modification, wood becomes gradually darker as temperature rises and time is prolonged [20,21,22,23]; the process of darkening happens in a uniform manner in the entire volume [24,25,26]. Changes caused by heat are important for species whose natural colour is grey and unattractive, such as poplar, or trees whose colour is not uniform, such as, e.g., black locust [27]. Thanks to thermal modification, it is possible to achieve wood with a light to dark brown colour, usually able to imitate and substitute the exotic species [6,28]. Moreover, in the case of thermally modified wood, less paints and lacquers can be used—and these substances are usually produced on the basis of solvents [29]. This is an important factor in view of environmental protection, as it allows us to limit or eliminate entirely these kinds of finish materials.

High modification temperatures cause a series of changes in the chemical properties of wood [30]. A series of reactions and chemical processes take place [31,32], the most important being hemicelluloses degradation, thermal plastification of cell walls, changes in lignin structure—guaiacyl and syringyl forms, cross-linking between polysaccharide macroparticles and between polysaccharides and lignin, and increases in cellulose crystallinity [33]. The change of wood colour to a darker colour results from the degradation of hemicelluloses and lignin—as well as their conversion into extractive chromophores [34,35].

Mass loss (ML) is one of the most important parameters described in the modification process [36,37,38]. Structure decomposition starts from about 150 °C. Hemicelluloses are degraded first, then cellulose, and the most resistant is lignin. Using an atmosphere with inert gases increases temperature resistance of natural wood. Mass loss is an ideal surrogate response to the severity of the treatment, resulting from the combination of the processing time and temperature. Changes in colour variables can be presented as functions of ML for inter-species comparisons. González-Peña and Hale [39] showed a negative curvilinear relationship between ΔL* and ML in beech, Norway spruce, and Scots pine wood after thermal modification in nitrogen atmosphere. Strong, curvilinear positive relationships were observed between the ML and ΔE in the three species. Higher treatment temperature and shorter reaction times led to similar ΔE with lower temperatures and longer processing times at equivalent levels of ML.

The modification of wood in nitrogen atmosphere is a process in the phase of research and implementation of results to the industrial phase, which determines the actions undertaken within the scope of optimisation of technological parameters. The aim of the study was to determine the impact of temperature and time of modification, and the interaction between them, on the colour of black poplar wood. It is expected that the research will contribute to the broadening of knowledge on the modification of fast-growing wood species, such as—among others—black poplar, in the context of its possible use as an alternative material to replace exotic wood.

## 2. Materials and Methods

### 2.1. Black Poplar Wood Samples

The modification was carried out on black poplar (*Populus nigra* L.) wood from a forest in Poland (Eastern part of the Mazovian province, State Forest District Sokołów Podlaski). It was solid wood of 40-year-old poplars. The trees had a diameter at breast height (DBH) up to 0.5 m and a mean growth ring width greater than 5 mm. The wood used in the study had no defects. The density of poplar wood determined according to the ISO 13061-2:2014 standard [40], at 0% moisture content, was 375 ± 38 kg·m^−3^. Wood moisture content was measured according to ISO 13061-1:2014 [41]. The dimensions of the samples used for modification were as follows: 20 mm (radial), 20 mm (tangential), and 300 mm (longitudinal). The surfaces of the wood samples were finished by planing. The control group was unmodified wood.

### 2.2. Black Poplar Thermal Modification in Nitrogen Atmosphere and Its Chemical Composition

The process of thermal modification of black poplar wood was carried out in nitrogen atmosphere. The modification was performed in an 0.25 m^3^ chamber (Explo Solutions sp. z o. o., Warsaw, Poland). The modification chamber was adjusted to working in pressures between −1 and 1 atm and was equipped with forced air circulation. The device was controlled by a computer, with the option to carry out a technological process composed of 8 steps and lasting up to 168 h.

**Figure 1:**
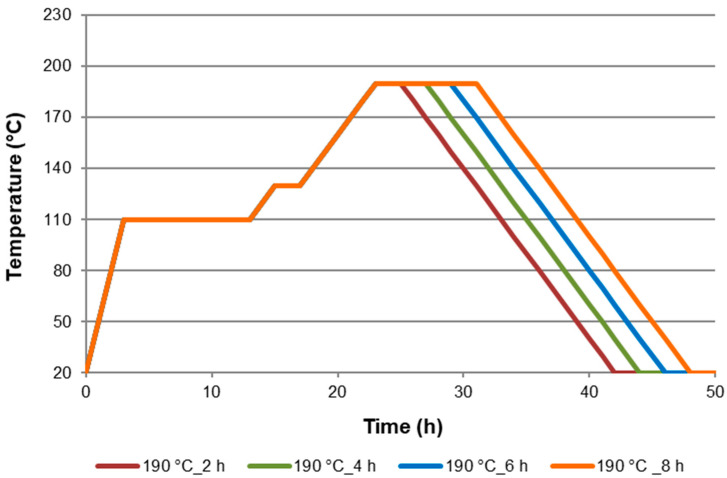
Poplar wood modification process in nitrogen atmosphere at 190 °C within 2 h, 4 h, 6 h, and 8 h.

The modification of black poplar wood was carried out at the temperatures of 160 °C, 170 °C, 180 °C, 190 °C, 200 °C, and 220 °C. The modification times (for each of the modification temperatures) were 2 h, 4 h, 6 h, and 8 h. Each variant of modification was performed on 30 samples, each of them characterised by a similar average density. The individual modification variants were carried out with a predefined time-temperature settings programme. The first stage of modification consisted of drying the wood for 10 h at a temperature of 110 °C. The next stage consisted of slowly heating the wood (10 °C/1 h) until reaching the temperature of 130 °C, and drying it for 2 h. The subsequent stage of modification consisted of reaching the target temperature (10 °C/1 h), and later the thermal processing of the material took place by continuing to heat the wood at the constant target temperature for the specified time. The last stage consisted in cooling the material by switching off the heating. The selected thermal modification programmes applied in the study are presented in Figure 1.

The mass loss (ML) was expressed as a percentage of the initial mass of totally dry wood. The ML was calculated according to Equation (1), where $m_{0}$ is the mass of the oven-dried wood (g), and $m_{m}$ is the mass of the oven-dried wood after thermal modification (g):

(1) $$\mathrm{ML}=\frac{m_{0}-{\;m}_{m}}{m_{0}}\times 100\;\left(\%\right).$$

Chemical compositions of non-modified and selected variants of thermally modified black poplar were determined according to the procedure presented in the Bytner et al. study [12]. Black poplar wood was subjected to chemical tests after modification at temperatures of 160 °C and 190 °C for 2 h and 6 h; and at 220 °C for 2 h.

### 2.3. Determination of Wood Colour Parameters

The parameters of wood colour were specified on the basis of the CIE L*a*b* model, where colour is described as the achromatic L* component and two chromatic components: a* and b*. The lightness L*, the chromatic coordinate on the red–green axis a*, and the chromatic coordinate on the yellow–blue axis b* were determined. The CIE L*a*b* model is one of the most frequently used to specify the colour of wood [42,43,44,45]. Wood colour parameters were measured in two points on the surface, in two sections (radial and tangential) for 15 samples from each modification. Measurements were made at a distance of 10 cm of the cross section of each sample.

In total, results were gathered from 30 measurement points from each section for non-modified wood, and for each modification variant. The device used to measure colour parameters was a SPEKTROMASTER 565-D (ERICHSEN GmbH & Co. KG, Hemer, Germany). Measurements were made with a D65 illuminant and a 10° standard observer. The total colour difference ΔE was determined in accordance with ISO 7724-3:1984 [46].

### 2.4. Statistical Analysis

The statistical analysis of results was carried out with the use of STATISTICA Version-12 software from StatSoft, Inc. (TIBCO Software Inc., Palo Alto, CA, USA). The analysis was based on the *t*-test or ANOVA (Fischer’s *F*-test), with a significance level (*p*) of 0.050. On the basis of the sum of squares (SS), we calculated the percent impact of the analysed factors (temperature and time of modification), the so-called Factor Influence on the colour parameters and total colour change (ΔE) of black poplar wood thermally modified in nitrogen atmosphere. The control group consisted of wood that did not undergo modification.

## 3. Results

**Figure 2:**
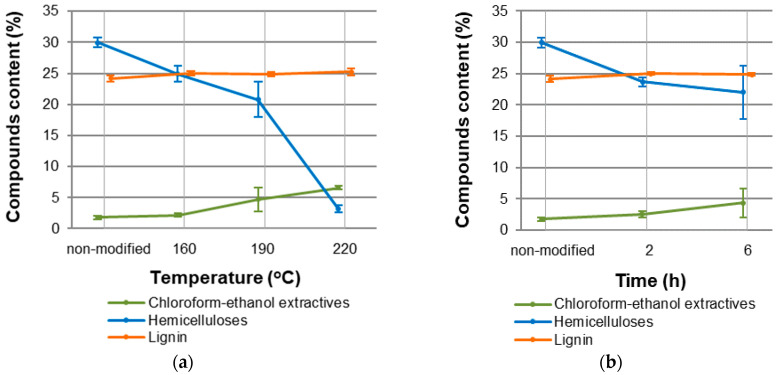
Chemical composition of non-modified and thermally modified in nitrogen atmosphere black poplar depends on (**a**) temperature modification, and (**b**) time modification.

As a result of thermal modification, black poplar wood underwent a mass loss (Table 1) caused by the degradation of structural wood components, mostly hemicelluloses (Figure 2a,b). Bytner et al. [12] showed that the share of hemicelluloses in non-modified black poplar wood amounted to ca. 30%. After a modification in 160 °C for 2 h, the share of hemicelluloses amounted to ca. 24%, and after the modification in 220 °C for 2 h, the share amounted to only 3%, so it was 10 times lower than in non-modified black poplar wood. The percent change in wood mass was more significant at higher temperatures and in longer times of the process. The mass loss (ML) of black poplar wood in the analysed modification conditions was in the range between 0.7% (±0.4%) and 14.0% (±1.1%). According to Bal [47], the ML after thermal modification of pine wood in nitrogen atmosphere amounted to 0.8%, 1.3%, and 2.9%, respectively, for the modification temperatures of 180 °C, 200 °C, and 220 °C and modification time of 2.5 h. At the same time, it must be noted that pine has a smaller hemicellulose content than deciduous species [48]. The degree of chemical changes in the wood can be expressed by mass loss, and it can be observed organoleptically as a change in colour. The relation between mass loss and colour change was analysed, among others, by González-Peña and Hale [39], and Olarescu and Campean [49].

**Figure 3:**
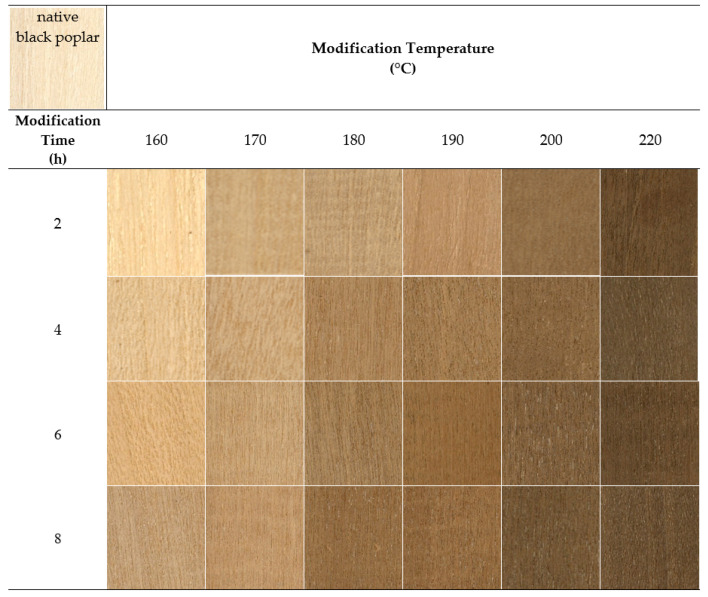
Black poplar wood before and after thermal modification in nitrogen atmosphere.

Figure 3 presents images of the tangential section of black poplar wood samples after thermal modification in nitrogen. Under the influence of the modification, black poplar wood changed to a darker colour, and the changes were more intense in higher temperatures and longer times of modification. From a practical point of view, it is important to note that the colour of thermally modified black poplar wood was similar to the colour of many exotic wood species. An improvement in the aesthetic value of wood results in better usability, which can lead to its higher competitive potential in the market of wood materials.

The values of lightness L* for black poplar wood in its radial and tangential sections are presented in Table 2. The L* value for non-modified (native) black poplar wood in its radial and tangential sections amounted to 80.9 (±4.3) and 79.4 (±4.3), respectively, and the differences were not statistically significant (Table 3). On the other hand, after the modification, the L* parameter value in the radial section was higher by 2% to 8% (depending on the temperature and time of modification) than the L* in the tangential section, and the differences were statistically significant. The only exception was observed for black poplar wood modified at 160 °C for 2 h. Most probably, the differences in the L* values in the different sections resulted from the presence of rays, in spite of their small number and size in the wood of black poplar. In the radial section, the rays have the form of “non-continuous bands”, and in the tangential section they can be seen as very thin “strands” with wedge-shaped ends. Black poplar wood contains 8–13 rays per tangential mm. They are not visible macroscopically, because there are exclusively uniseriate, and what is more, aggregate rays are absent. Rays are composed of a single cell type (homocellular). The height of large rays can reach 500 µm [50,51]. Moreover, the differences in the cell layout (vessels and fibres) in the radial and tangential section can cause a different angle of light ray reflection and result in different L* values being measured. Higher L* values for the radial section than for the tangential section in tested hardwood species were also observed by Nishino et al. [52]. On the other hand, Nasir et al. [53] proved that western hemlock (*Tsuga heterophylla*) wood becomes darker with increasing heat treatment (ThermoWood^®^ process) intensity, with no significant difference between lightness of the flatsawn and quartersawn samples.

A reduction of the L* parameter value caused by the modification process was observed, and it depended on the temperature and time of modification. The trends were similar for both kinds of section. The L* parameter for the radial section of black poplar wood modified at 160 °C and 220 °C for 2 h was, correspondingly, lower by 10% and 42% than for non-modified wood. On the other hand, after 8 h of processing at 160 °C, the differences were similar and amounted to ca. 10%, and at the temperature of 220 °C, the L* values were lower by 48%. On the other hand, the L* parameter for the tangential section of black poplar wood modified at 160 °C and 220 °C for 2 h was lower by 11% and 44% than in non-modified wood. After 8 h of processing at 160 °C, the differences were similar and amounted to ca. 11%, and at the temperature of 220 °C, the L* values were lower by 50%. Overall, it can be concluded that L* decreased slowly for the modification at 160 °C and 170 °C, the dynamics of changes was greater at 180 °C and 190 °C, and changed a lot at 200 °C and 220 °C.

A similar decrease in L* values was observed by Brito et al. [54]. The authors noticed changes in the L* parameter of yellow poplar after thermal modification in air atmosphere, carried out at the temperatures of 180 °C, 200 °C, and 220 °C for 2.5 h, by 18%, 27%, and 54%, respectively, compared to non-modified wood. González-Peña and Hale [39] tested the influence of thermal modification in nitrogen on the colour of, among others, beech wood. The authors concluded that, in case of beech, L* decreased slowly only for the treatment at 190 °C, colour conversion was fast for the treatment at 210 °C, and almost instantaneous at 230 °C and at 245 °C. It was also noticed that the profile of L* reduction was similar to that of weight loss in thermally modified wood and presumably manifested the chemical stabilisation of thermal modification upon increased lengths of heat treatment. The changes in beech wood colour lightness had the strongest correlation with hemicelluloses. Similar conclusions were drawn by Sundqvist and Morén [55], who pointed out that the phenomenon of wood darkening is related mainly to hemicellulose degradation caused by high temperatures.

As to the section (radial or tangential), statistically significant differences were observed between the L* values for the modification variants, depending on modification time and temperature (Table 3). Only in very few cases were no significant differences found (marked as ns). Therefore, it confirmed that the temperature and time of modification (in the entire analysed range) have an influence on the L* parameter of black poplar thermally modified in nitrogen atmosphere.

The values of the a* parameter in the radial and tangential section of black poplar wood modified in nitrogen atmosphere are presented in Table 4. The a* values for non-modified black poplar wood in its radial and tangential sections amounted to 4.0 (±1.6) and 4.3 (±1.6), respectively, and the differences were not statistically significant (Table 5). The statistical analyses carried out revealed that most of the analysed variants of modification did not represent significant differences between the a* parameter of the radial and tangential section.

In general, it can be concluded that the modification of black poplar wood changed its colour towards red. The increase in the a* parameter of black poplar wood was noticed after modification in the temperature range 160 °C–200 °C. The longer the time of modification, the greater were the changes (Table 4). Generally, it can be concluded that the a* parameter values after modification at 200 °C were two times higher than in the case of non-modified black poplar wood. However, after the modification at 220 °C (independently of the time of the process), the a* values started to drop. This resulted from the fact that after reaching a certain maximum, the a* parameter started to decrease in value. Similar results were shown by Barcík et al. [24] for birch wood modified with the ThermoWood^®^ method. After 5 h of modification at 210 °C, the authors observed a* values around 9.09, and at 240 °C, a* amounted to 6.70. Kučerová et al. [56] indicated that during the modification of fir wood under atmospheric pressure in the presence of air, the maximum value of the a* parameter amounted to 10.4 and was achieved at the temperature of 220 °C and time of 1 h.

After modification, depending on the analysed processing parameters, the a* value for the radial and tangential section of black poplar wood fell within the range between ca. 6 and ca. 10. González-Peña and Hale [39] tested the influence of thermal modification in nitrogen on the colour of beech wood and noticed that a* bears a complex, non-linear profile. The authors observed that for beech wood, the maximum a* value of ca. 6 was achieved at the temperature of 190 °C and time of modification of 8 h. The changes in the a* parameter in beech had the strongest correlation with lignin. According to Chen et al. [57], the increase in the a* parameter is due to oxidation, condensation, and degradation of wood components. Such substances as lignin and certain extractive substances can condensate and create byproducts, resulting in a higher intensity of the red in the wood. Marcon et al. [58] explained that the variance in the a* parameter results from many chemical reactions taking place at different times of the treatment process. This is the result of, first, the oxidation reaction, followed afterwards by a degradation of the constitutive hemicellulose polymers. At the temperature of ca. 120 °C, hemicelluloses are decomposed, creating formic and acetic acids that accelerate and additionally catalyse the hydrolysis of hemicelluloses and amorphic cellulose [59,60]. Lignin undergoes a loss of methoxy groups, and the researchers claim that there is an increase in crosslink density [61].

For non-modified black poplar wood, the b* parameter values amounted to ca. 18 (Table 6). There were no statistically significant differences between the b* values for radial and tangential sections. This also applied to most of the black poplar modification variants tested in the study (Table 7). It is worth noting that in the tested sections (radial or tangential), in many cases, there were no statistically significant differences (marked as ns) between the b* parameter values of black poplar wood modified at temperatures between 160 °C and 190 °C. On this basis, it was stated that the modification in this temperature range does not significantly influence the variation of the chromatic coordinate on the yellow–blue axis.

In general, it can be concluded that the modification of black poplar wood changed its colour towards yellow. The increase in the b* parameter of black poplar wood was noticed after modification in the temperature range 160 °C–190 °C (independently of the duration of modification). On the other hand, after modification at the temperature of 200 °C, the b* values started to decrease, and after modification at the temperature of 220 °C (depending on the duration of modification), they were similar to or lower than the b* of non-modified black poplar wood. Similar results were described by Kučerová et al. [56], in relation to silver fir thermally modified under atmospheric pressure in the presence of air.

The highest value of the b* parameter for black poplar wood in the radial and tangential section was achieved after treatment in 190 °C for 4 h, and it amounted to 24.3 (±1.1) and 23.7 (±1.0), respectively (Table 6). For the radial section, the maximum value of the b* parameter for black poplar wood was higher by 39% than in the case of non-modified wood. In case of the b* parameter measured for the tangential section, this difference amounted to 31%. Gao et al. [25] modified fast-growing poplar wood under vacuum heat treatment, using the temperatures 140 °C, 160 °C, 180 °C, and 200 °C and times of 1 h, 2 h, and 3 h. The highest value of the b* parameter the authors observed was for 160 °C and 3 h, and it was higher by 23% than the b* of non-modified wood. González-Peña and Hale [39] tested the influence of thermal modification in nitrogen on the colour of beech wood and also noticed that b* had a non-linear profile. After reaching the maximum value, b* values started to go down together with longer times of thermal modification. It was observed that for beech wood, the maximum b* value, depending on the temperature of modification, was between 18 and 22. Moreover, the relationship between the chemical changes and colour modifications was also analysed, and it was concluded that the change in the b* parameter was strongly correlated with glucuronoxylan in beech. Research conducted by Kubovský et al. [62] confirmed that thermal modification causes chemical changes in polysaccharides and also in lignin. The factors accountable for wood becoming yellow include primarily lignin and its derivatives, i.e., quinones and stilbenes [63,64,65]. As the temperature of treatment rises, the amount of hemicelluloses that undergo degradation increases, and, consequently, the lignin content in wood and the susceptibility of wood to a change in colour towards yellow also increase. Yellowing is the main change in the colour of wood, being an effect of lignin photodegradation [66,67,68].

Wood colour change has a great potential to predict thermal modification quality [39,69]. Table 8 presents the ΔE values for the radial and tangential section of black poplar wood modified in nitrogen atmosphere. The change in colour had a similar character for both sections. The modification process caused an increase in the ΔE value, which depended on the temperature and duration of modification. The differences of ΔE between the radial and tangential sections were not statistically significant (Table 9), which indicates a “uniform” colour in both sections. The statistical analysis also confirms that there were significant differences in the colour of black poplar wood after modification, using the set process parameters.

In general, it can be concluded that the changes in colour of black poplar wood after modification at the temperature of 160 °C and 170 °C were similar, and the dynamics of changes was greater after modification at the temperature of 180 °C. The highest value of ΔE was observed for modification temperature of 220 °C and time of 8 h, and it amounted to 38.9 (±4.8) and 40.0 (±4.8) for the radial and tangential section, correspondingly (Table 8). Nguyen et al. [70] noticed similar changes for poplar wood (*Populus alba*) modified in nitrogen atmosphere. After the modification of poplar wood at 220 °C for 8 h, the ΔE amounted to 41.76. Gurleyen et al. [71] obtained similar results for poplar wood (*Populus deltoides*) modified with the ThermoWood^®^ method, where ΔE amounted to 40.72 for the temperature of 212 °C and time of 2 h. Barcík et al. [24] indicated that after the thermal treatment of birch wood (carried out according to the ThermoWood^®^ process) at the temperature of 240 °C and time of 5 h, the maximum value of ΔE reached 17.77.

According to the evaluation criteria of overall colour change ΔE, high colour changes take place when ΔE falls in the range between 6 and 12, and above the value of 12 they are considered two different colours [72]. On this basis, it can be concluded that the modification at the temperature of 160 °C and 170 °C resulted in high colour changes of black poplar wood. In the remaining variants of thermal modification in nitrogen, the change of colour was so large (ΔE above 12) that it can be considered that black poplar wood had a different colour. The change in colour can be explained by the generation of coloured products of hemicellulose degradation, extractive substances, and lignin [55,56,73]. It is proposed that ΔE in thermally modified wood originates from chemical changes in the main wood polymers, more so in lignin than in polysaccharides, due to the darkening of the lignin itself. This was associated with the generation of chromophoric groups, mainly the increase in carbonyl groups appearing in the Fourier transform infrared spectra of lignin between 1710 and 1600 cm^−1^, particularly the emergence of quinone species [39]. The formation of oxidation products such as quinones is considered to be the reason for the colour change [20].

**Figure 4:**
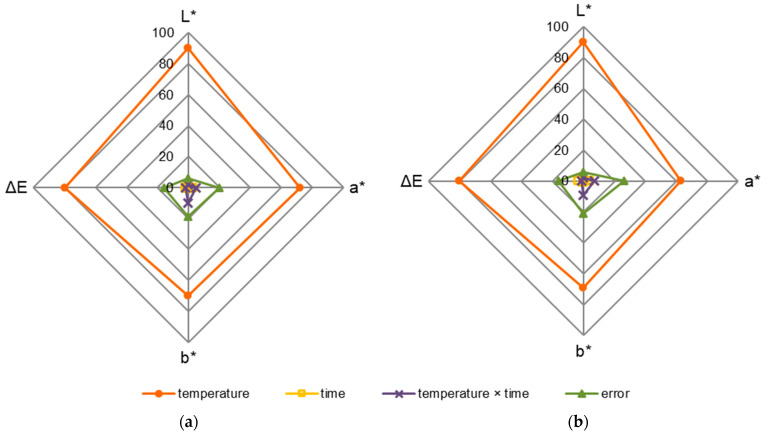
The percent influence of temperature, modification time, and interaction between temperature and modification time on the colour parameters (L*, a*, b*) and total colour changes ΔE on the (**a**) radial and (**b**) tangential section of thermally modified black poplar.

Figure 4 shows the statistical evaluation of the influence of the factors on the colour parameters of black poplar wood. The percentage effect of the factors was estimated on the basis of the sum of squares—SS (ANOVA, Fisher’s *F*-test, *p* ≤ 0.050). The statistical analyses that were carried out clearly indicate that the temperature of modification had the greatest influence on the colour parameters of black poplar wood, and as a result—the value of ΔE (Figure 4a,b). Independently of the tested section, the temperature of modification was responsible for the L* parameter values in about 90%, and the a* and b* values in about 70%. The influence of the duration of modification on the tested colour parameters was small, i.e., below 4%. The influence of the interaction between modification temperature and time on the colour parameters was also low, i.e., below 10%.

In the case of ΔE, the influence of temperature was at a level of ca. 80%. On the other hand, the influence of time and the interaction between temperature and time of modification was small, i.e., below 3%. The obtained error values indicate that there are also other factors that determine the values of colour components, and they also influence the change in colour, i.e., wood species, age, extractive content, soil water [71,74]. There are many factors that influence species selection for usage [75]. One of them is the colour of the wood. For this reason, the presented observations are of a utilitarian nature.

## 4. Conclusions

As a result of thermal modification in nitrogen, the colour of black poplar wood underwent significant changes. The character of changes of the individual colour components, that is, L*, a*, and b*, was different and it was correlated with the treatment process parameters. On the basis of the study, the following can be clearly concluded:

- Black poplar wood is very susceptible to colour change as a result of thermal modification in nitrogen, independently of the section (radial, tangential).
- Modification at the temperatures of 160 °C and 170 °C caused high colour changes of black poplar wood. After modification at the temperatures of 180 °C and higher, the change in colour was so large (ΔE above 12) that it can be considered that black poplar wood already had a different colour.
- The observed changes did not have a linear character in case of the a* and b* parameters, whose maximum values were observed at modification temperatures of 200 °C and 190 °C, respectively. In case of L* and ΔE, the biggest changes happened at the highest temperature and the longest time of modification (220 °C, 8 h).

Colour of thermally modified black poplar is linked with chemical changes. Hemicelluloses are the most susceptible to thermal degradation, then chloroform–ethanol extractives. No statistically significant changes were observed for the lignin content in the black poplar wood before and after the modification process.

## Figures and Tables

**Table 1:** Mass loss of black poplar wood thermally modified in nitrogen atmosphere; ±(SD).

Modification Time (h)	Modification Temperature (°C)					
	160	170	180	190	200	220
	Mass Loss (%)					
2	0.9 ± 0.5	0.8 ± 0.1	0.8 ± 0.4	0.8 ± 0.4	4.0 ± 0.3	6.8 ± 0.9
4	0.7 ± 0.4	0.8 ± 0.1	1.9 ± 0.1	2.6 ± 0.1	5.4 ± 0.3	12.0 ± 0.9
6	0.7 ± 0.4	0.9 ± 0.5	2.2 ± 0.5	2.1 ± 0.7	5.8 ± 0.9	12.6 ± 0.9
8	0.9 ± 0.4	1.1 ± 0.1	3.0 ± 0.2	3.5 ± 0.6	7.1 ± 1.0	14.0 ± 1.1

**Table 2:** Values of the L* parameter of black poplar wood thermally modified in nitrogen atmosphere; ±(SD).

Poplar Wood	Radial Section						Tangential Section					
Native	80.9 ± 4.3						79.4 ± 4.3					
Modified	Modification Temperature (°C)						Modification Temperature (°C)					
Modification Time (h)	160	170	180	190	200	220	160	170	180	190	200	220
2	72.9 ± 3.9	73.1 ± 2.0	68.6 ± 3.6	65.8 ± 2.0	56.3 ± 2.8	46.9 ± 2.5	71.0 ± 3.7	69.5 ± 2.6	66.5 ± 3.4	62.3 ± 3.2	54.5 ± 1.9	44.8 ± 2.6
4	75.3 ± 2.7	70.9 ± 3.1	66.1 ± 2.8	61.1 ± 2.1	51.8 ± 3.2	44.8 ± 2.8	73.6 ± 2.2	65.8 ± 2.8	61.4 ± 2.0	58.0 ± 1.5	49.5 ± 2.2	41.0 ± 2.0
6	73.0 ± 2.6	71.1 ± 1.6	64.7 ± 3.2	59.4 ± 2.3	53.4 ± 2.5	43.0 ± 1.7	71.4 ± 3.2	68.6 ± 2.8	60.6 ± 2.1	57.1 ± 2.4	51.2 ± 2.3	39.8 ± 2.4
8	73.7 ± 2.9	70.3 ± 2.2	59.9 ± 2.4	58.4 ± 2.1	51.1 ± 2.7	42.3 ± 2.0	70.6 ± 2.4	66.4 ± 2.9	56.6 ± 2.1	56.3 ± 3.0	48.3 ± 2.5	39.8 ± 2.3

**Table 3:** Statistical analysis of the L* results (*t*-test, *p* ≤ 0.050, *—significant dependence, ns—no significant dependence).

Radial vs. Tangential	Native Wood	160 °C_2 h	160 °C_4 h	160 °C_6 h	160 °C_8 h	170 °C_2 h	170 °C_4 h	170 °C_6 h	170 °C_8 h	180 °C_2 h	180 °C_4 h	180 °C_6 h	180 °C_8 h	190 °C_2 h	190 °C_4 h	190 °C_6 h	190 °C_8 h	200 °C_2 h	200 °C_4 h	200 °C_6 h	200 °C_8 h	220 °C_2 h	220 °C_4 h	220 °C_6 h	220 °C_8 h	
**Native Wood**	**ns**	*	*	*	*	*	*	*	*	*	*	*	*	*	*	*	*	*	*	*	*	*	*	*	*	**Radial**
**160 °C_2 h**	*	**ns**	*	ns	ns	ns	*	*	*	*	*	*	*	*	*	*	*	*	*	*	*	*	*	*	*	
**160 °C_4 h**	*	*	*	*	*	*	*	*	*	*	*	*	*	*	*	*	*	*	*	*	*	*	*	*	*	
**160 °C_6 h**	*	ns	*	*	ns	ns	*	*	*	*	*	*	*	*	*	*	*	*	*	*	*	*	*	*	*	
**160 °C_8 h**	*	ns	*	ns	*	ns	*	*	*	*	*	*	*	*	*	*	*	*	*	*	*	*	*	*	*	
**170 °C_2 h**	*	ns	*	*	ns	*	*	*	*	*	*	*	*	*	*	*	*	*	*	*	*	*	*	*	*	
**170 °C_4 h**	*	*	*	*	*	*	*	ns	ns	*	*	*	*	*	*	*	*	*	*	*	*	*	*	*	*	
**170 °C_6 h**	*	*	*	*	*	ns	*	*	ns	*	*	*	*	*	*	*	*	*	*	*	*	*	*	*	*	
**170 °C_8 h**	*	*	*	*	*	*	ns	*	*	*	*	*	*	*	*	*	*	*	*	*	*	*	*	*	*	
**180 °C_2 h**	*	*	*	*	*	*	ns	*	ns	*	*	*	*	*	*	*	*	*	*	*	*	*	*	*	*	
**180 °C_4 h**	*	*	*	*	*	*	*	*	*	*	*	ns	*	ns	*	*	*	*	*	*	*	*	*	*	*	
**180 °C_6 h**	*	*	*	*	*	*	*	*	*	*	ns	*	*	ns	*	*	*	*	*	*	*	*	*	*	*	
**180 °C_8 h**	*	*	*	*	*	*	*	*	*	*	*	*	*	*	*	ns	*	*	*	*	*	*	*	*	*	
**190 °C_2 h**	*	*	*	*	*	*	*	*	*	*	ns	*	*	*	*	*	*	*	*	*	*	*	*	*	*	
**190 °C_4 h**	*	*	*	*	*	*	*	*	*	*	*	*	*	*	*	*	*	*	*	*	*	*	*	*	*	
**190 °C_6 h**	*	*	*	*	*	*	*	*	*	*	*	*	ns	*	ns	*	ns	*	*	*	*	*	*	*	*	
**190 °C_8 h**	*	*	*	*	*	*	*	*	*	*	*	*	ns	*	*	ns	*	*	*	*	*	*	*	*	*	
**200 °C_2 h**	*	*	*	*	*	*	*	*	*	*	*	*	*	*	*	*	*	*	*	*	*	*	*	*	*	
**200 °C_4 h**	*	*	*	*	*	*	*	*	*	*	*	*	*	*	*	*	*	*	*	*	*	*	*	*	ns	
**200 °C_6 h**	*	*	*	*	*	*	*	*	*	*	*	*	*	*	*	*	*	*	*	*	*	*	*	*	*	
**200 °C_8 h**	*	*	*	*	*	*	*	*	*	*	*	*	*	*	*	*	*	*	*	*	*	*	*	*	*	
**220 °C_2 h**	*	*	*	*	*	*	*	*	*	*	*	*	*	*	*	*	*	*	*	*	*	*	*	*	*	
**220 °C_4 h**	*	*	*	*	*	*	*	*	*	*	*	*	*	*	*	*	*	*	*	*	*	*	*	*	*	
**220 °C_6 h**	*	*	*	*	*	*	*	*	*	*	*	*	*	*	*	*	*	*	*	*	*	*	*	*	ns	
**220 °C_8 h**	*	*	*	*	*	*	*	*	*	*	*	*	*	*	*	*	*	*	ns	*	*	*	*	ns	*	
	**Tangential**																									

**Table 4:** Values of the a* parameter of black poplar wood thermally modified in nitrogen atmosphere; ±(SD).

Poplar Wood	Radial Section						Tangential Section					
Native	4.0 ± 1.6						4.3 ± 1.6					
Modified	Modification Temperature (°C)						Modification Temperature (°C)					
Modification Time (h)	160	170	180	190	200	220	160	170	180	190	200	220
2	5.9 ± 0.6	7.0 ± 0.7	7.7 ± 0.5	8.1 ± 0.5	9.5 ± 0.7	8.5 ± 0.6	6.1 ± 0.8	7.6 ± 0.5	7.8 ± 0.6	8.1 ± 0.5	9.3 ± 0.4	8.5 ± 0.7
4	6.0 ± 0.7	7.2 ± 0.6	8.2 ± 0.6	9.4 ± 0.6	9.6 ± 0.5	8.3 ± 0.5	6.3 ± 0.6	7.5 ± 0.5	8.4 ± 0.5	9.4 ± 0.5	9.5 ± 0.6	8.4 ± 0.5
6	6.5 ± 0.7	7.2 ± 0.4	8.4 ± 0.5	8.9 ± 0.5	9.5 ± 0.5	8.3 ± 0.4	6.8 ± 0.7	7.5 ± 0.9	8.6 ± 0.6	8.9 ± 0.4	9.2 ± 0.5	8.0 ± 0.5
8	6.7 ± 0.6	7.6 ± 0.5	9.4 ± 0.5	9.4 ± 0.5	9.6 ± 0.5	8.1 ± 0.5	7.1 ± 0.6	8.0 ± 0.5	9.4 ± 0.7	9.2 ± 0.6	9.6 ± 0.7	8.0 ± 0.7

**Table 5:** Statistical analysis of the a* results (*t*-test, *p* ≤ 0.050, *—significant dependence, ns—no significant dependence).

Radial vs. Tangential	Native Wood	160 °C_2 h	160 °C_4 h	160 °C_6 h	160 °C_8 h	170 °C_2 h	170 °C_4 h	170 °C_6 h	170 °C_8 h	180 °C_2 h	180 °C_4 h	180 °C_6 h	180 °C_8 h	190 °C_2 h	190 °C_4 h	190 °C_6 h	190 °C_8 h	200 °C_2 h	200 °C_4 h	200 °C_6 h	200 °C_8 h	220 °C_2 h	220 °C_4 h	220 °C_6 h	220 °C_8 h	
**Native Wood**	**ns**	*	*	*	*	*	*	*	*	*	*	*	*	*	*	*	*	*	*	*	*	*	*	*	*	**Radial**
**160 °C_2 h**	*	**ns**	ns	*	*	*	*	*	*	*	*	*	*	*	*	*	*	*	*	*	*	*	*	*	*	
**160 °C_4 h**	*	ns	**ns**	*	*	*	*	*	*	*	*	*	*	*	*	*	*	*	*	*	*	*	*	*	*	
**160 °C_6 h**	*	*	*	**ns**	ns	*	*	*	*	*	*	*	*	*	*	*	*	*	*	*	*	*	*	*	*	
**160 °C_8 h**	*	*	*	ns	*	ns	*	*	*	*	*	*	*	*	*	*	*	*	*	*	*	*	*	*	*	
**170 °C_2 h**	*	*	*	*	*	*	ns	ns	*	*	*	*	*	*	*	*	*	*	*	*	*	*	*	*	*	
**170 °C_4 h**	*	*	*	*	*	ns	**ns**	ns	*	*	*	*	*	*	*	*	*	*	*	*	*	*	*	*	*	
**170 °C_6 h**	*	*	*	*	ns	ns	ns	**ns**	*	*	*	*	*	*	*	*	*	*	*	*	*	*	*	*	*	
**170 °C_8 h**	*	*	*	*	*	*	*	*	*	ns	*	*	*	*	*	*	*	*	*	*	*	*	*	*	*	
**180 °C_2 h**	*	*	*	*	*	ns	*	ns	ns	**ns**	*	*	*	*	*	*	*	*	*	*	*	*	*	*	*	
**180 °C_4 h**	*	*	*	*	*	*	*	*	*	*	**ns**	ns	*	ns	*	*	*	*	*	*	*	*	ns	ns	ns	
**180 °C_6 h**	*	*	*	*	*	*	*	*	*	*	ns	**ns**	*	*	*	*	*	*	*	*	*	ns	ns	ns	ns	
**180 °C_8 h**	*	*	*	*	*	*	*	*	*	*	*	*	**ns**	*	ns	*	ns	ns	*	ns	ns	*	*	*	*	
**190 °C_2 h**	*	*	*	*	*	*	*	*	ns	ns	*	*	*	**ns**	*	*	*	*	*	*	*	*	ns	ns	ns	
**190 °C_4 h**	*	*	*	*	*	*	*	*	*	*	*	*	ns	*	**ns**	*	ns	ns	ns	ns	ns	*	*	*	*	
**190 °C_6 h**	*	*	*	*	*	*	*	*	*	*	*	*	*	*	*	**ns**	*	*	*	*	*	*	*	*	*	
**190 °C_8 h**	*	*	*	*	*	*	*	*	*	*	*	*	ns	*	ns	*	**ns**	ns	ns	ns	ns	*	*	*	*	
**200 °C_2 h**	*	*	*	*	*	*	*	*	*	*	*	*	ns	*	ns	*	ns	**ns**	ns	ns	ns	*	*	*	*	
**200 °C_4 h**	*	*	*	*	*	*	*	*	*	*	*	*	ns	*	ns	*	*	ns	**ns**	ns	ns	*	*	*	*	
**200 °C_6 h**	*	*	*	*	*	*	*	*	*	*	*	*	ns	*	ns	*	ns	ns	*	*	ns	*	*	*	*	
**200 °C_8 h**	*	*	*	*	*	*	*	*	*	*	*	*	ns	*	ns	*	*	ns	ns	*	**ns**	*	*	*	*	
**220 °C_2 h**	*	*	*	*	*	*	*	*	*	*	ns	ns	*	*	*	*	*	*	*	*	*	**ns**	ns	ns	ns	
**220 °C_4 h**	*	*	*	*	*	*	*	*	*	*	ns	ns	*	*	*	*	*	*	*	*	*	ns	**ns**	ns	ns	
**220 °C_6 h**	*	*	*	*	*	*	*	*	ns	ns	*	*	*	ns	*	*	*	*	*	*	*	*	*	*	ns	
**220 °C_8 h**	*	*	*	*	*	*	*	*	ns	ns	*	*	*	ns	*	*	*	*	*	*	*	*	*	ns	**ns**	
	**Tangential**																									

**Table 6:** Values of the b* parameter of black poplar wood thermally modified in nitrogen atmosphere; ±(SD).

Poplar Wood	Radial Section						Tangential Section					
Native	17.5 ± 1.1						18.1 ± 1.0					
Modified	Modification Temperature (°C)						Modification Temperature (°C)					
Modification Time (h)	160	170	180	190	200	220	160	170	180	190	200	220
2	20.7 ± 1.2	21.2 ± 1.3	22.3 ± 1.1	22.5 ± 1.1	23.5 ± 0.9	18.1 ± 1.2	20.9 ± 1.4	21.2 ± 1.0	21.0 ± 1.3	21.9 ± 1.3	23.2 ± 1.0	18.5 ± 1.5
4	20.9 ± 1.5	23.2 ± 0.8	23.0 ± 1.1	24.3 ± 1.1	21.3 ± 1.1	17.2 ± 1.6	20.6 ± 1.1	23.0 ± 1.1	22.7 ± 0.6	23.7 ± 1.0	21.0 ± 1.2	16.4 ± 1.3
6	21.3 ± 1.4	21.6 ± 1.2	22.8 ± 1.0	23.3 ± 1.1	22.3 ± 1.2	16.4 ± 0.9	21.3 ± 1.2	21.8 ± 1.2	22.6 ± 1.2	22.7 ± 0.8	21.5 ± 1.3	15.4 ± 1.5
8	20.9 ± 0.8	22.3 ± 1.5	24.1 ± 1.0	23.2 ± 0.7	20.8 ± 1.2	16.1 ± 0.9	20.9 ± 1.2	22.2 ± 1.5	23.6 ± 1.3	22.9 ± 1.1	20.5 ± 1.4	15.5 ± 1.6

**Table 7:** Statistical analysis of the b* results (*t*-test, *p* ≤ 0.050, *—significant dependence, ns—no significant dependence).

Radial vs. Tangential	Native Wood	160 °C_2 h	160 °C_4 h	160 °C_6 h	160 °C_8 h	170 °C_2 h	170 °C_4 h	170 °C_6 h	170 °C_8 h	180 °C_2 h	180 °C_4 h	180 °C_6 h	180 °C_8 h	190 °C_2 h	190 °C_4 h	190 °C_6 h	190 °C_8 h	200 °C_2 h	200 °C_4 h	200 °C_6 h	200 °C_8 h	220 °C_2 h	220 °C_4 h	220 °C_6 h	220 °C_8 h	
**Native Wood**	*	*	*	*	*	*	*	*	*	*	*	*	*	*	*	*	*	*	*	*	*	*	*	*	*	**Radial**
**160 °C_2 h**	*	**ns**	ns	ns	ns	ns	*	*	*	*	*	*	*	*	*	*	*	*	ns	*	ns	*	*	*	*	
**160 °C_4 h**	*	ns	**ns**	ns	ns	ns	*	ns	*	*	*	*	*	*	*	*	*	*	ns	*	ns	*	*	*	*	
**160 °C_6 h**	*	ns	*	**ns**	ns	ns	*	ns	*	*	*	*	*	*	*	*	*	*	ns	*	ns	*	*	*	*	
**160 °C_8 h**	*	ns	ns	ns	**ns**	ns	*	*	*	*	*	*	*	*	*	*	*	*	ns	*	ns	*	*	*	*	
**170 °C_2 h**	*	ns	*	ns	ns	**ns**	*	ns	*	*	*	*	*	*	*	*	*	*	ns	*	ns	*	*	*	*	
**170 °C_4 h**	*	*	*	*	*	*	**ns**	*	*	*	ns	ns	*	*	*	ns	ns	ns	*	*	*	*	*	*	*	
**170 °C_6 h**	*	*	*	ns	*	*	*	**ns**	ns	*	*	*	*	*	*	*	*	*	ns	*	*	*	*	*	*	
**170 °C_8 h**	*	*	*	*	*	*	*	ns	**ns**	ns	*	ns	*	ns	*	*	*	*	*	ns	*	*	*	*	*	
**180 °C_2 h**	*	*	*	*	*	*	*	ns	ns	**ns**	*	*	*	ns	*	*	*	*	*	ns	*	*	*	*	*	
**180 °C_4 h**	*	*	*	*	*	*	ns	*	ns	*	**ns**	ns	*	ns	*	ns	ns	ns	*	*	*	*	*	*	*	
**180 °C_6 h**	*	*	*	*	*	*	ns	*	ns	ns	ns	**ns**	*	ns	*	ns	ns	*	*	ns	*	*	*	*	*	
**180 °C_8 h**	*	*	*	*	*	*	ns	*	*	*	*	*	**ns**	*	ns	*	*	*	*	*	*	*	*	*	*	
**190 °C_2 h**	*	*	*	*	*	*	*	ns	ns	ns	*	ns	*	**ns**	*	*	*	*	*	ns	*	*	*	*	*	
**190 °C_4 h**	*	*	*	*	*	*	*	*	*	*	*	*	ns	*	*	*	*	*	*	*	*	*	*	*	*	
**190 °C_6 h**	*	*	*	*	*	*	ns	*	ns	*	ns	ns	*	*	*	*	ns	ns	*	*	*	*	*	*	*	
**190 °C_8 h**	*	*	*	*	*	*	ns	*	*	*	ns	ns	*	*	*	ns	**ns**	ns	*	*	*	*	*	*	*	
**200 °C_2 h**	*	*	*	*	*	*	ns	*	*	*	*	*	ns	*	ns	*	ns	**ns**	*	*	*	*	*	*	*	
**200 °C_4 h**	*	ns	ns	ns	ns	ns	*	*	*	*	*	*	*	*	*	*	*	*	**ns**	*	ns	*	*	*	*	
**200 °C_6 h**	*	ns	*	ns	ns	ns	*	ns	*	ns	*	*	*	ns	*	*	*	*	ns	*	*	*	*	*	*	
**200 °C_8 h**	*	ns	ns	*	ns	*	*	*	*	*	*	*	*	*	*	*	*	*	ns	*	**ns**	*	*	*	*	
**220 °C_2 h**	*	*	*	*	*	*	*	*	*	*	*	*	*	*	*	*	*	*	*	*	*	**ns**	*	*	*	
**220 °C_4 h**	*	*	*	*	*	*	*	*	*	*	*	*	*	*	*	*	*	*	*	*	*	*	*	*	*	
**220 °C_6 h**	*	*	*	*	*	*	*	*	*	*	*	*	*	*	*	*	*	*	*	*	*	*	*	*	ns	
**220 °C_8 h**	*	*	*	*	*	*	*	*	*	*	*	*	*	*	*	*	*	*	*	*	*	*	*	ns	**ns**	
	**Tangential**																									

**Table 8:** Values of the colour differences ΔE of black poplar wood thermally modified in nitrogen atmosphere; ±(SD).

Modification Time (h)	Radial Section						Tangential Section					
	Modification Temperature (°C)						Modification Temperature (°C)					
	160	170	180	190	200	220	160	170	180	190	200	220
2	9.4 ± 3.0	9.4 ± 3.7	13.5 ± 4.3	16.5 ± 4.7	25.9 ± 4.2	34.4 ± 4.3	9.3 ± 3.2	11.0 ± 4.0	14.0 ± 5.0	18.1 ± 5.5	26.1 ± 4.2	34.9 ± 6.0
4	7.3 ± 3.4	12.4 ± 3.0	17.5 ± 3.8	21.7 ± 5.2	29.9 ± 6.1	36.4 ± 5.5	6.9 ± 2.9	13.8 ± 2.8	19.1 ± 4.5	22.8 ± 4.6	30.6 ± 5.3	38.7 ± 5.1
6	9.5 ± 3.1	11.1 ± 3.2	17.7 ± 4.9	22.9 ± 4.7	28.5 ± 6.0	38.2 ± 4.8	8.6 ± 3.3	11.8 ± 3.9	19.9 ± 5.3	23.3 ± 4.7	28.9 ± 4.9	39.9 ± 5.6
8	8.6 ± 3.4	12.4 ± 3.4	22.7 ± 4.9	23.9 ± 4.8	30.5 ± 4.8	38.9 ± 5.2	9.7 ± 3.8	13.3 ± 3.1	24.1 ± 4.7	24.3 ± 5.6	31.8 ± 4.6	40.0 ± 4.8

**Table 9:** Statistical analysis of the ΔE results (*t*-test, *p* ≤ 0.050, *—significant dependence, ns—no significant dependence).

Radial vs. Tangential	160 °C_2 h	160 °C_4 h	160 °C_6 h	160 °C_8 h	170 °C_2 h	170 °C_4 h	170 °C_6 h	170 °C_8 h	180 °C_2 h	180 °C_4 h	180 °C_6 h	180 °C_8 h	190 °C_2 h	190 °C_4 h	190 °C_6 h	190 °C_8 h	200 °C_2 h	200 °C_4 h	200 °C_6 h	200 °C_8 h	220 °C_2 h	220 °C_4 h	220 °C_6 h	220 °C_8 h	
**160 °C_2 h**	**ns**	*	ns	ns	ns	*	*	*	*	*	*	*	*	*	*	*	*	*	*	*	*	*	*	*	**Radial**
**160 °C_4 h**	*	**ns**	*	ns	*	*	*	*	*	*	*	*	*	*	*	*	*	*	*	*	*	*	*	*	
**160 °C_6 h**	ns	*	**ns**	ns	ns	*	ns	*	*	*	*	*	*	*	*	*	*	*	*	*	*	*	*	*	
**160 °C_8 h**	ns	*	ns	**ns**	ns	*	*	*	*	*	*	*	*	*	*	*	*	*	*	*	*	*	*	*	
**170 °C_2 h**	ns	*	*	ns	**ns**	*	ns	*	*	*	*	*	*	*	*	*	*	*	*	*	*	*	*	*	
**170 °C_4 h**	*	*	*	*	*	**ns**	ns	ns	ns	*	*	*	*	*	*	*	*	*	*	*	*	*	*	*	
**170 °C_6 h**	*	*	*	*	ns	*	**ns**	ns	*	*	*	*	*	*	*	*	*	*	*	*	*	*	*	*	
**170 °C_8 h**	*	*	*	*	*	ns	*	**ns**	ns	*	*	*	*	*	*	*	*	*	*	*	*	*	*	*	
**180 °C_2 h**	*	*	*	*	*	ns	ns	ns	**ns**	*	*	*	*	*	*	*	*	*	*	*	*	*	*	*	
**180 °C_4 h**	*	*	*	*	*	*	*	*	*	**ns**	ns	*	*	*	*	*	*	*	*	*	*	*	*	*	
**180 °C_6 h**	*	*	*	*	*	*	*	*	*	ns	**ns**	*	ns	*	*	*	*	*	*	*	*	*	*	*	
**180 °C_8 h**	*	*	*	*	*	*	*	*	*	*	*	**ns**	*	ns	ns	ns	*	*	*	*	*	*	*	*	
**190 °C_2 h**	*	*	*	*	*	*	*	*	*	ns	ns	*	**ns**	*	*	*	*	*	*	*	*	*	*	*	
**190 °C_4 h**	*	*	*	*	*	*	*	*	*	*	*	ns	*	**ns**	ns	ns	*	*	*	*	*	*	*	*	
**190 °C_6 h**	*	*	*	*	*	*	*	*	*	*	*	ns	*	ns	**ns**	ns	*	*	*	*	*	*	*	*	
**190 °C_8 h**	*	*	*	*	*	*	*	*	*	*	*	ns	*	ns	ns	**ns**	ns	*	*	*	*	*	*	*	
**200 °C_2 h**	*	*	*	*	*	*	*	*	*	*	*	ns	*	*	*	ns	**ns**	*	ns	*	*	*	*	*	
**200 °C_4 h**	*	*	*	*	*	*	*	*	*	*	*	*	*	*	*	*	*	**ns**	ns	ns	*	*	*	*	
**200 °C_6 h**	*	*	*	*	*	*	*	*	*	*	*	*	*	*	*	*	*	*	**ns**	ns	*	*	*	*	
**200 °C_8 h**	*	*	*	*	*	*	*	*	*	*	*	*	*	*	*	*	*	*	*	**ns**	*	*	*	*	
**220 °C_2 h**	*	*	*	*	*	*	*	*	*	*	*	*	*	*	*	*	*	*	*	*	**ns**	ns	*	*	
**220 °C_4 h**	*	*	*	*	*	*	*	*	*	*	*	*	*	*	*	*	*	*	*	*	*	**ns**	ns	ns	
**220 °C_6 h**	*	*	*	*	*	*	*	*	*	*	*	*	*	*	*	*	*	*	*	*	*	ns	**ns**	ns	
**220 °C_8 h**	*	*	*	*	*	*	*	*	*	*	*	*	*	*	*	*	*	*	*	*	*	ns	ns	**ns**	
	**Tangential**																								

## Data Availability

The data presented in this study are available upon request from the corresponding author.
